# Preoperative cranial computed tomography in pediatric cochlear implantation: clinical necessity or optional step?

**DOI:** 10.3389/fneur.2026.1981222

**Published:** 2026-10-23

**Authors:** L. Fries, F. Everad, G. Magradze, F. Deffner, A. Aschendorff, S. Arndt, M. C. Ketterer

**Affiliations:** Department of Otorhinolaryngology, Faculty of Medicine, Medical Center, University of Freiburg, Freiburg im Breisgau, Germany

**Keywords:** cranial CT children, cranial MRI children, pediatric cochlear implant, pediatric cochlear implantation, pediatric cranial imaging, preoperative imaging cochlear implantation, temporal bone CT children

## Abstract

**Objectives:**

Due to growing evidence regarding the risks of ionizing radiation, especially in children, some centers now perform cochlear implantation (CI) without a preoperative computed tomography (CT) scan, relying solely on magnetic resonance imaging (MRI). This study evaluates the necessity and safety of omitting CT scans before bilateral CI in children.

**Methods:**

A retrospective analysis of 164 children, implanted bilaterally between 2018 and 2024 and aged under 10 years at the time of either simultaneous or second CI, were included. The impact of preoperative imaging on duration of surgery and perioperative complications was analyzed. The surgical procedure was compared between cases with and without a preoperative CT scan and analyzed for surgeons in training and experienced surgeons.

**Results:**

105 children were implanted with a preoperative CT scan and 59 without a CT scan. The duration of bilateral CI surgery was 122.5 (±41.4) min for procedures [CI without CT: 122.5 (±41.4) min; CI with CT: 116.1 (±33.84) min; (*p* = 0.4861)] and anesthesia [CI without CT: 237.2 (±47.38) min; CI with CT: 226.3 (±41.50) min; (*p* = 0.1352)] did not differ significantly between the groups, irrespective of the experience of the surgeons. Seven cases showed relevant malformations visible on MRI, such as large vestibular aqueduct syndrome, which were relevant during the surgery. Minor and major complications occurred rarely in both cohorts but more frequently in patients without CT scan (without CT: 10.2% (6/59); with CT: 4.8% (5/105); *p* = 0.2052; risk difference [RD], 5.4%; 95% CI, −6.3 to 14.3%). Intraoperative X-ray imaging revealed no cases of electrode tip fold-over, and no patients developed postoperative complete or incomplete facial nerve palsy.

**Conclusion:**

In summary, an MRI-based imaging strategy without routine preoperative CT may be safe and sufficient in appropriately selected pediatric CI candidates, with additional CT reserved for patients in whom clinical or MRI findings suggest relevant bony abnormalities, complex inner ear malformations, or syndromic conditions. This approach reduces unnecessary radiation exposure and the use of healthcare resources, while avoiding redundant diagnostic information from both imaging modalities, without compromising surgical safety or outcomes.

## 1 Introduction

The prevalence of pediatric hearing impairment is estimated at 1–4%, with SNHL affecting approximately 0.1% of children and adolescents in Germany (1). Owing to widespread newborn hearing screening programs, severe to profound sensorineural hearing loss (SNHL) is often diagnosed shortly after birth, necessitating early hearing rehabilitation (2). For both pediatric and adult patients with SNHL, CI and consequent postoperative hearing rehabilitation represents the standard of care (3). In children, CI before speech development is associated with better audiological outcomes and may result in hearing performance comparable to that of normally hearing children for up to 10 years after implantation. Showing comparable performance to that of normally hearing children up to 10 years after implantation (4, 5). Given that age of implantation is a crucial factor in the benefits of CI for children, CI is increasingly being performed on young infants without increased risk of perioperative morbidity and complications as shown in the literature (4, 6–8).

Prior to CI, patients require an extensive evaluation of their auditory system, including preoperative imaging, to diagnose the etiology, determine candidacy for CI and to provide realistic expectations for the patients and their families. In addition to audiological testing, which includes objective measurements, cranial imaging is conducted using computed tomography (CT) scans of the temporal bone or cranial magnetic resonance imaging (MRI) (9). The purpose of preoperative imaging is to identify contraindications to CI and anatomical abnormalities that may influence candidacy or surgical planning. This is particularly relevant in children with SNHL, in whom inner ear malformations have been reported in up to 30% of cases (10). Typically, patients receive an MRI and a temporal bone CT scan before CI; however, additional CT scans are less common in children (9, 11). MRI allows for soft tissue evaluation, particularly of the cochlear nerve, retrocochlear and intracranial pathologies and possibly intracochlear fibrosis or malformations as well as fluid in the scale of the cochlea. In contrast, CT provides detailed visualization of the bony anatomy, including cochlear morphology and critical structures relevant to surgical planning and the risk of perioperative complications (12–15). In addition, preoperative temporal bone CT can be used with planning software such as OTOPLAN® (CAScination AG, Bern, Switzerland, in cooperation with MED-EL, Innsbruck, Austria) to assess cochlear dimensions and plan electrode insertion (16). However, CT exposes children to ionizing radiation, with particular concern regarding radiation-related risks in young patients (17–19). Consequently, some centers have adopted an MRI-only strategy for pediatric CI, reserving temporal bone CT for selected cases with specific risk factors, such as craniofacial anomalies or previous temporal bone trauma (11, 20). These studies suggest that omitting routine preoperative CT does not increase perioperative complications or duration of surgery (11, 20, 21). Emerging photon-counting detector CT (PCD-CT) technology may further reduce radiation exposure while maintaining high spatial resolution (22). Furthermore, the potential financial burden of extended preoperative imaging should be considered, particularly with increasing CI numbers and healthcare costs (23, 24). Although dedicated temporal bone CT acquisition is rapid, performing CT only subsequently after MRI may require an additional diagnostic visit and potentially a second session of anesthesia, increasing the overall burden and costs. Thus, the potential benefit of avoiding radiation with an MRI-based strategy must be weighed against the additional anesthesia and resource requirements associated with subsequent CT (11, 20, 25). With the increasing number of CI in young children, evidence-based guidance is needed to determine whether preoperative CT provides clinically relevant information beyond MRI that influences surgical planning or intraoperative management (21).

The aim of the present study is to evaluate the necessity and safety of omitting CT scans before (bilateral) CI in children. We aimed to determine whether additional CT findings not detected on MRI had a clinically relevant impact on surgical planning or intraoperative management. The study intends to compare children who underwent preoperative CT scans with those who did not, in terms of peri- and postoperative complications, duration of surgery, and length of hospital stay, following a transition in our institution’s imaging protocol in 2021. The purpose of this study is to provide implications for the future, offer evidence for a uniform recommendation on whether to perform additional CT scan and define criteria for patients requiring extended preoperative imaging. We hypothesize that omission of the preoperative temporal bone CT is not associated with increased perioperative complications or prolonged duration of surgery.

## 2 Methods

A retrospective analysis of 164 children was performed in our department of Otorhinolaryngology, Head and Neck surgery, at the Implant Center of the University Hospital Freiburg. Patients implanted bilaterally between 2018 and 2024 and aged under 10 years at the time of either simultaneous or second CI, were included from our database. Following an institutional preoperative imaging protocol change in 2021, preoperative imaging prior to CI transitioned from routine combined MRI and CT to an MRI-only strategy, meaning patient allocation was determined by the timeline of this policy update rather than randomization. The primary outcomes of this study were the cumulative duration of surgery and the incidence of perioperative complications, specifically focusing on cerebrospinal fluid (CSF) leakage and facial nerve palsy. Secondary outcomes included the total duration of anesthesia and the length of hospitalization. Additionally, the study evaluated imaging-specific endpoints by comparing the diagnostic and surgical impact of the two preoperative imaging strategies (combined MRI and CT versus an MRI-only approach). These outcomes were further analyzed based on the surgical experience level, comparing procedures performed by surgeons in training (<5 years of experience) and with those completed by experienced surgeons (≥5 years of high-volume experience at our institution). The statistical analysis was done with Prism 7-software (GraphPad Software, Inc., La Jolla, CA, United States). Normal distribution was tested with D Agostino and Pearson normality test. Significance level of *p < 0.05* was set for all statistical analysis. We used the nonparametric Mann–Whitney test and Fisher’s exact test for statistical analysis of the duration of surgery, anesthesia and hospitalization as well as the complication rate and difference in the imaging strategies. The study was approved by the local ethics committee in accordance with the Declaration of Helsinki (Washington, 2002) (ethics committee approval number: 406/19, amendment number: 240275).

## 3 Results

### 3.1 Demographics

A total of 164 children implanted bilaterally between 2018 and 2024 were included. The mean age at bilateral simultaneous CI or at first CI was 24.64 ± 19.15 months. Children who underwent preoperative CT imaging were significantly older (mean age, 26.44 ± 20.03 months) than the children who did not (mean age, 21.44 ± 17.18 months; *p* < 0.0352). In the overall study cohort, 81 patients were female and 83 were male. Among children without a preoperative CT scan, 16.9% (10/59) underwent sequential CI, whereas 83.1% (49/59) received simultaneous CI. In children with a preoperative CT scan, sequential CI was performed in 36.2% (38/105), and simultaneous CI in 63.8% (67/105) of cases.

### 3.2 Causes for SNHL

Figure 1 summarizes the etiologies of hearing loss in patients with and without preoperative CT. Congenital hearing loss was the most common etiology in both cohorts [58.1% (61/105) with CT vs. 66.1% (39/59) without CT]. Infectious and genetic etiologies were similarly distributed between groups, whereas inner ear malformations were more frequently identified in the CT cohort [21.9% (23/105) vs. 1.7% (1/59)] in the cohort without CT. Detailed etiological findings, including specific infectious, genetic, and anatomical diagnoses, are provided in Supplementary Table 1.

**Figure 1:**
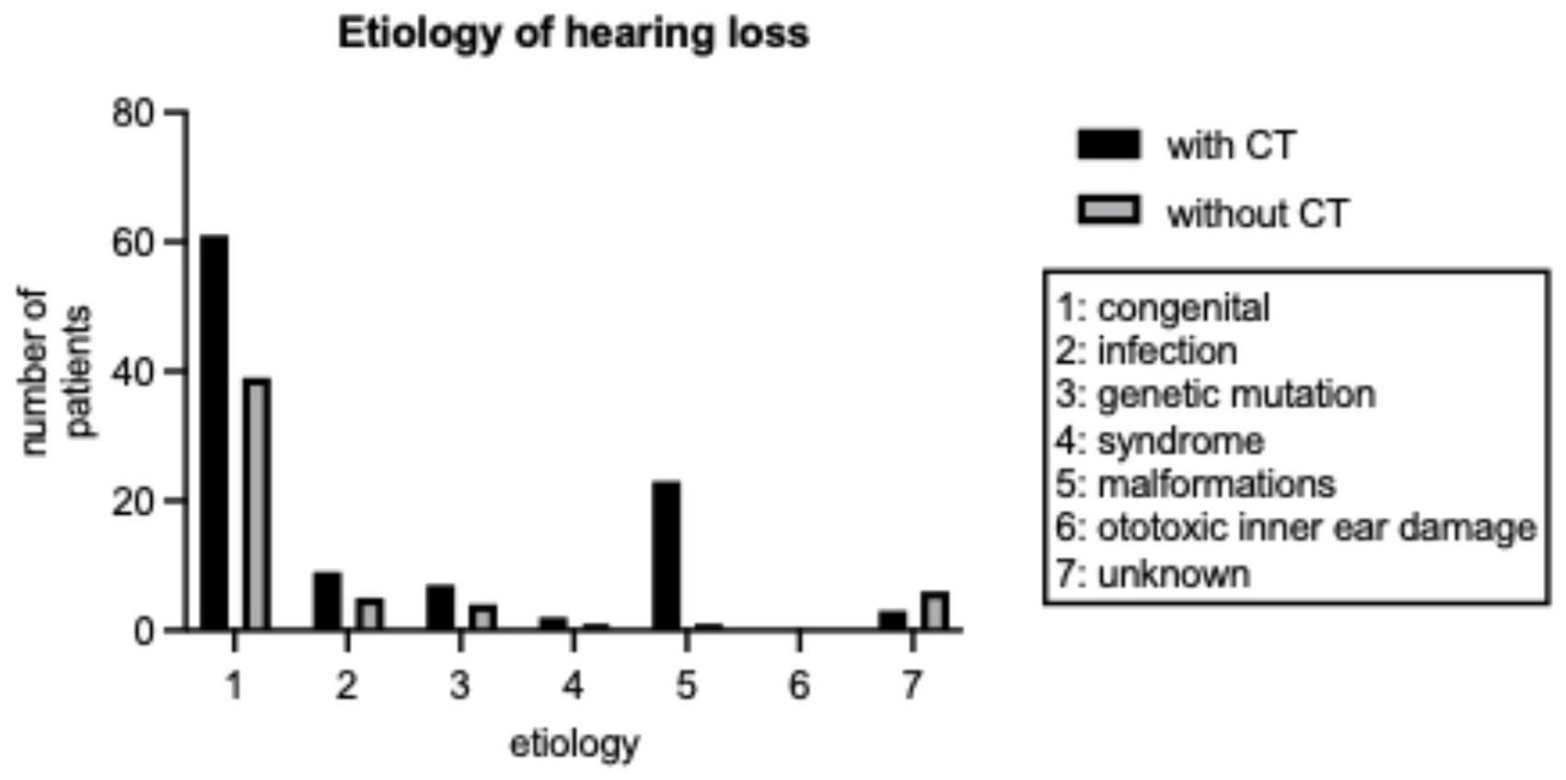
Etiology of hearing loss for cohorts with and without preoperative CT scan.

### 3.3 Radiological evaluation

A change in the institutional preoperative imaging protocol was initiated in 2021, transitioning from routine combined MRI and CT imaging to an MRI-only strategy prior to CI. The first implantation without a preoperative CT scan in our cohort was performed in April 2020. During the transitional year 2021, twelve patients received MRI alone, whereas seven patients continued to undergo combined MRI and CT prior to surgery. Before March 2022, 86% (104/121) of patients underwent combined MRI and CT imaging. Following full implementation of the revised protocol in March 2022, MRI was used as the sole preoperative imaging modality.

Of the 164 children included in the study, 59 underwent CI without preoperative CT imaging, whereas 105 received an additional preoperative CT scan. Intraoperative X-ray was performed in all patients to detect a potential tip fold-over of the electrode array, regardless of whether preoperative CT imaging had been performed (Table 1).

**Table 1:** Descriptive study cohort details comparing patients with and without preoperative CT scans (IQR = interquartile range; SD = standard deviation, min = minimum, max = maximum).

	CT	No CT	*p*
Patients (*n*)	105	59	
Age (months) [mean, (SD; range)]	26.44 (±20.03;8–110)	21.44 (±17.18; 6–90)	0.0352
Sex (male/female), *n* (%)	53 (50.5%)/52 (49.5%)	30 (50.8%)/29 (49.2%)	0.999
CI surgery (sequential/simultaneous), *n* (%)	38 (36.2%)/67 (63.8%)	10 (16.9%)/49 (83.1%)	0.0119

Additionally, all 164 patients in the study cohort underwent preoperative cMRI. In the 59 patients without preoperative CT scans, 83.0% (49/59) of MRIs were unremarkable, while 17.0% (10/59) showed abnormalities (see Table 2). Of these, only one pathology—dysplasia of the vestibule and inferior and lateral semicircular canals—affected surgery, causing resistance during insertion without prolonging operative time.

**Table 2:** Pathologic findings in preoperative imaging in patients with cranial MRI solely.

MRI finding	Relevance to CI surgery	Surgical impact
Arachnoidal cyst	No	No surgical impact
Rathke’s cleft cyst of the pituitary gland	No	No surgical impact
Focal cortical dysplasia, Mega cisterna magna, Microcephaly	No	No surgical impact
Dysplasia of the vestibule and inferior and lateral semicircular canals	Yes	Yes (Flattening of the lateral semicircular canal, resistance during insertion)
Benign lesion at foramen magnum	No	No surgical impact
Subcortical lesion postcentral gyrus	No	No surgical impact
Pineal cyst	No	No surgical impact
Benign enlargement of the subarachnoid spaces	No	No surgical impact
Microcephaly, Polymicrogyria	No	No surgical impact
Bilaterally hyperintensities along the medial lemniscus	No	No surgical impact

Among the 105 patients who additionally underwent preoperative CT, MRI demonstrated no abnormalities or malformations in 72 patients (68.6%), whereas pathological findings were identified in 33 patients (31.4%). Of these findings, only 23 were considered potentially relevant to CI surgery and are summarized in Table 3. However, only seven findings had an impact on the surgical procedure. Intraoperative perilymphatic leakage (gusher) occurred in six procedures: five in patients with documented bilateral large vestibular aqueduct (LVA) and one in a patient with bilateral cochlear dysplasia accompanied by dysplasia of the superior semicircular canals (Table 3). In one other patient with malformed apical turns of the cochlear anterior-inferior cochleostomy was performed. Of the potentially relevant findings identified in 21 cases on temporal bone CT scans, 11 were also detected on cMRI and 10 were not detected on cMRI (see Table 4). Two of the 23 potentially relevant findings were only detected on cMRI and not in additional performed CT scans (see Table 3). However, only one CT-only finding was associated with a modification of the surgical procedure, consisting of an anterior-inferior cochleostomy for malformed apical cochlear turns. Conversely, in two patients, cMRI revealed abnormalities that were not detected on CT scans, namely vestibular hypoplasia and a loop of the anterior inferior cerebellar artery contacting the vestibulocochlear nerve.

**Table 3:** Pathologic 23 findings in preoperative combined imaging with dual MRI and CT scan of the temporal bone which are potentially relevant during CI surgery (LVA = large vestibular aqueduct; IP = incomplete partition; CMV = cytomegalovirus; AICA = anterior inferior cerebellar artery).

MRI finding	CT finding	Relevance to CI surgery	Surgical impact
Malformed inner ear with absent posterior semicircular canals (bilateral), pineal cyst	Hypoplasia of posterior semicircular canals (bilateral)	Yes	No surgical impact
Bilateral LVA, malformed apical cochlear turns (compatible with IP-II)	Bilateral LVA	Yes	Anterior-inferior cochleostomy; perilymph leak (bilateral)
Bilateral LVA	Bilateral LVA	Yes	Anterior-inferior cochleostomy; perilymph leak (bilateral)
Bilateral LVA	Bilateral LVA	Yes	Anterior-inferior cochleostomy; perilymph leak (bilateral)
–	Suspected anterior semicircular canal dehiscence (left)	Yes	No surgical impact
–	Malformed bilateral apical cochlear turns	Yes	Anterior-inferior cochleostomy
–	Suspected bony dehiscence superior semicircular canals (bilateral)	Yes	No surgical impact
–	Bony dehiscence of anterior and posterior semicircular canals (bilateral)	Yes	No surgical impact
–	Bony dehiscence of posterior semicircular canals (bilateral)	Yes	No surgical impact
–	Bony dehiscence of anterior and lateral semicircular canals (bilateral)	Yes	No surgical impact
Bilateral LVA	Bilateral LVA	Yes	Anterior-inferior cochleostomy; perilymph leak (bilateral)
–	Suspected bony dehiscence of anterior and posterior semicircular canals (bilateral)	Yes	No surgical impact
Bilateral cochlear dysplasia with dysplasia of superior semicircular canals	Bilateral cochlear dysplasia with dysplasia of superior semicircular canals	Yes	Anterior-inferior cochleostomy; perilymph leak (bilateral) Extended coverage; Gusher with need of CI Revision
–	Hypoplasia of the semicircular canals	Yes	No surgical impact
Suspected complex middle/inner ear malformation, Cochlear Incomplete Partition Type I (IP-I).	Suspected complex middle/inner ear malformation, Cochlear Incomplete Partition Type I (IP-I).	Yes	No surgical impact
–	Posterior and superior semicircular canal dehiscence (bilateral)	Yes	No surgical impact
Arnold-Chiari I malformation with suspected hydrocephalus	Posterior semicircular canal dehiscence (bilateral)	Yes	No surgical impact
Bilateral LVA	Bilateral LVA	Yes	Anterior-inferior cochleostomy; perilymph leak (bilateral)
Vestibular hypoplasia (left), hypoplastic labyrinth (bilateral)	–	Yes	No surgical impact
AICA loop (left) contacting vestibulocochlear nerve	–	Yes	No surgical impact
–	Suspected bony dehiscence superior semicircular canals (bilateral)	Yes	No surgical impact
Aplasia of posterior semicircular canals (bilateral)	Aplasia of posterior semicircular canals (bilateral)	Yes	No surgical impact
Suspected bony dehiscence superior semicircular canals (bilateral)	Suspected bony dehiscence superior semicircular canals (bilateral)	Yes	No surgical impact

**Table 4:** Perioperative mortality and major complications as well as anesthesiological complications showed no significant differences in cases with and without CT scan.

	CT	No CT	*p*
Perioperative minor complications [%, (*n*)]*	4.8% (5/105)	10.2% (6/59)	ns (*p* = 0.2052)
Perioperative major complications [%, (*n*)]	0% (0/105)	0% (0/59)	ns (*p* > 0.9999)
Perioperative mortality [%, (*n*)]	0% (0/105)	0% (0/59)	ns (*p* > 0.9999)
Anesthesiological complications [%, (*n*)]**	6.7% (7/105)	in 8.5% (5/59)	ns (*p* = 0.7575)
Length of hospitalization (days, SD)	6.57 (2.31)	6.07 (1.95)	ns (*p* = 0.1903)

Perioperative minor complications were more frequent without CT scan without being statistically significant. *minor-complications: hematoma, wound infection, fever, vertigo, pain exacerbation, liquor leak; **Prolonged time to extubation, bradycardia, bronchial reaction, difficult intubation; ns, not significant; SD, standard deviation.

### 3.4 Cochlear implant surgery

The overall mean duration of surgery for bilateral CI across all patients was 118.4 min (±36.74). The mean duration of surgery for bilateral CI was 122.5 min (±41.4) for procedures without preoperative CT and 116.1 min (±33.84) for those with CT, with no statistically significant difference (*p* = 0.4861), as shown in Figure 2. After adjustment for age, duration of surgery for bilateral CI was 8.13 min shorter in the cohort with CT scan than in the cohort without CT scan, but this difference was not statistically significant (*β* = −8.13, 95% CI − 20.29 to 4.03, *p* = 0.189). Among surgeons in training, the duration of surgery was longer for surgeries performed without CT (184.4 min ± 42.64) and those with CT (159.3 min ± 89.79), although this difference was not statistically significant (*p* = 0.4818). Similarly, for experienced surgeons, duration of surgery was 111.3 min (±29.99) without CT and 114.9 min (±31.03) with CT, showing no significant difference (*p* = 0.5283) in Figure 3. Across all patients, the mean duration of anesthesia for bilateral CI was 230.2 min (±42.65). Procedures performed without preoperative CT tended to take slightly longer (237.2 ± 47.38 min) than those with CT (226.3 ± 41.50 min), although this difference was not statistically significant in the unadjusted analysis (*p* = 0.1352) (see Figure 4). However, after adjustment for age, anesthesia duration for bilateral CI was significantly shorter in patients who underwent preoperative CT compared with those who did not (*β* = −15.56 min, 95% CI − 30.07 to −1.05, *p* = 0.036). This difference indicates that age adjustment influenced the estimated association between preoperative CT and anesthesia duration, despite age itself not being significantly associated with anesthesia duration (*β* = 0.27 min/year, 95% CI − 0.09 to 0.63, *p* = 0.135).

**Figure 2:**
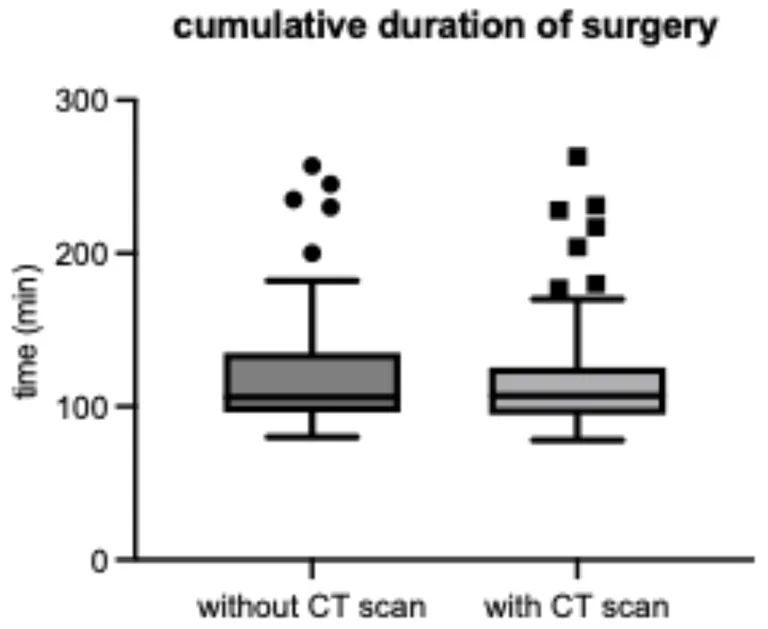
The cumulative duration of surgery did not significantly differ according to preoperative CT imaging (without CT: 122.5 ± 41.4 min; with CT: 116.1 ± 33.8 min; *p* = 0.4861).

**Figure 3:**
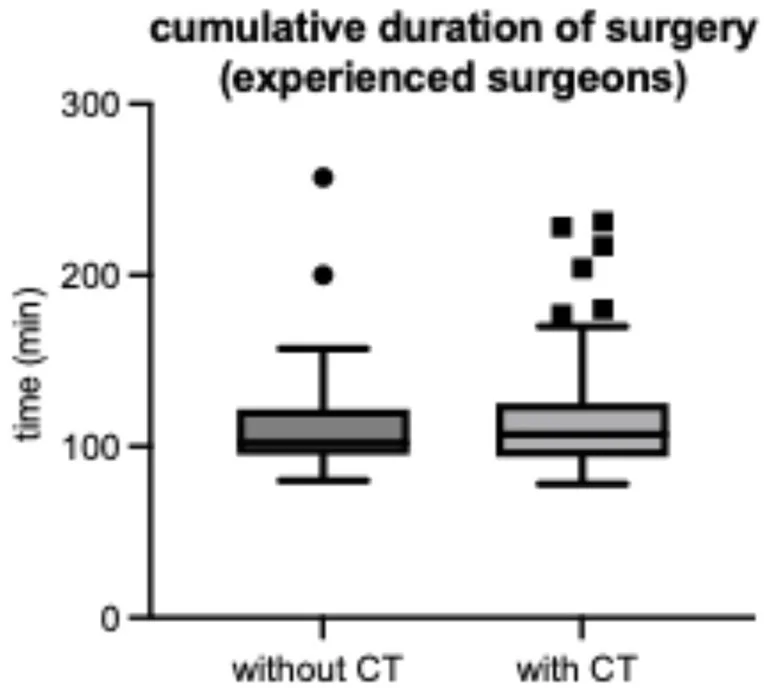
For experienced surgeons duration of surgery did also not differ significantly depending on preoperative CT imaging. (without CT: 111.3 min (±29.99); with CT: 114.9 min (±31.03); *p* = 0.5283).

**Figure 4:**
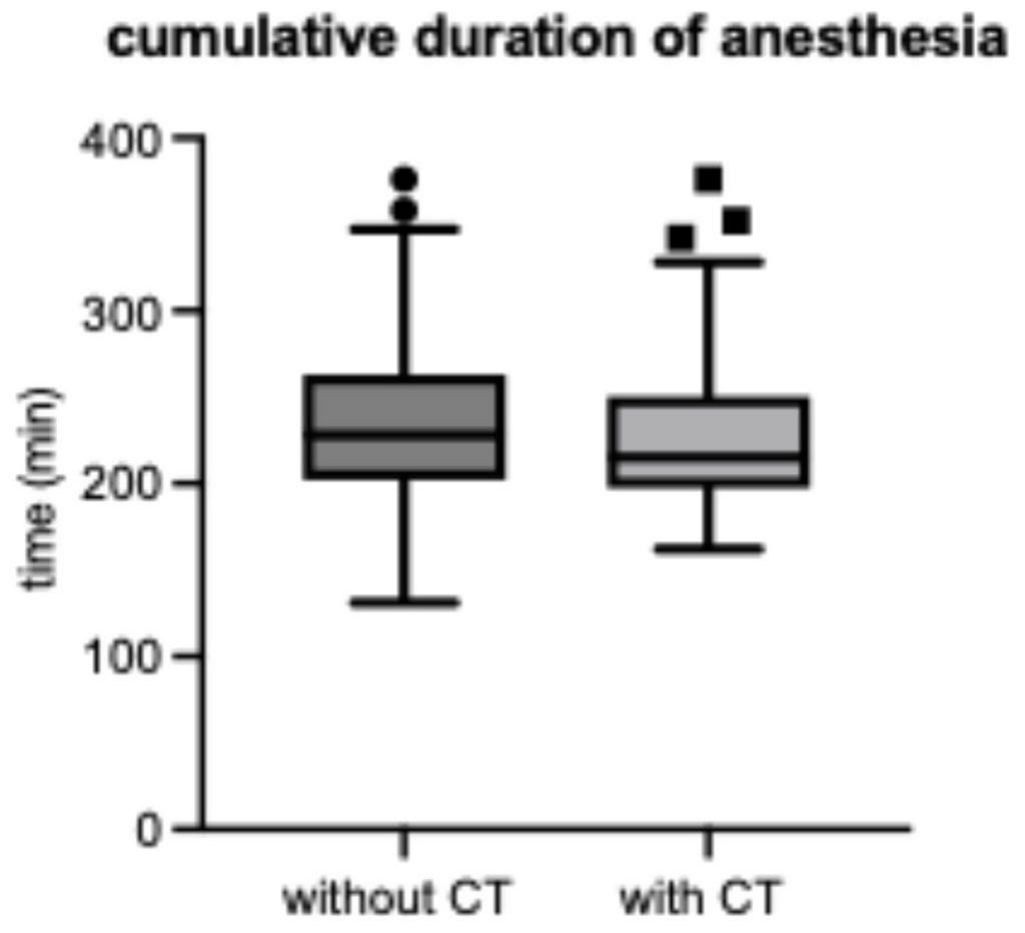
Procedures performed without preoperative CT tended to take slightly longer (237.2 ± 47.38 min) than those with CT (226.3 ± 41.50 min), but were not significant (*p* = 0.1352).

### 3.5 Perioperative and postoperative complications

Minor and major complications occurred rarely and did not differ significantly when compared between the patients with preoperative CT and the ones without (see Table 4). Fatal complications did not occur in any patient, irrespective of CT imaging (see Supplementary Table 1; *p* > 0.9999). No postoperative facial nerve palsy or paralysis occurred in either patients with or without a preoperative CT scan. No tip fold-over of the electrode array was seen in the intraoperative X-rays. As shown in Table 4, perioperative minor complications such as hematoma, wound infection, fever, vertigo, pain exacerbation and cerebrospinal fluid (CSF) leak were rare in both cohorts but occurred more frequent in patients without CT scan (without CT: 10.2% (6/59); with CT: 4.8% (5/105); *p* = 0.2052; risk difference [RD], 5.4%; 95% CI, −6.3 to 14.3%). One patient in each cohort experienced postoperative vertigo, which resolved spontaneously after several days. In one case of postoperative CSF leak, the patient had Johanson–Blizzard syndrome with cochlear dysplasia on CT imaging and required revision surgery with sealing of the cochleostomy 7 days after the initial CI. Postoperative wound infection occurred in three patients (two with preoperative CT imaging and one without). Two cases were treated with intravenous antibiotics, whereas one required revision surgery with partial CI explantation 22 days after the initial procedure, followed by successful reimplantation 4 months later. Postoperative fever occurred in three patients (two without CT and one with CT), and pain exacerbation was reported in one patient without CT imaging. A postoperative hematoma occurred in one patient without preoperative CT imaging and necessitated surgical revision with hematoma evacuation 7 days after CI. There was no significant difference in anesthesiologic complications between the two cohorts. Minor anesthetic events occurred in 6.7% (7/105) of patients with preoperative CT and in 8.5% (5/59) of those without CT, including prolonged time to extubating, bronchial reactions, and difficult intubation. Median hospital stay was 6 days in both groups, with no difference related to preoperative CT scans (see Table 4).

## 4 Discussion

Prior to CI, cranial imaging is fundamental to identify possible contraindications for surgery and plan the surgical procedure, which may require adjustments. CT scans are associated with ionizing radiation, while MRI scans necessitate sedation for infants and young children and incur a greater financial burden (17, 18, 23–25). Each imaging modality has its own disadvantages, which lead to variability in use across institutions (11). Combined CT and MRI imaging provides comprehensive data but introduces diagnostic redundancy, prolonged anesthesia, and radiation risks (11, 25, 26). While minimizing pediatric radiation exposure remains a priority (17–20), recent advances like PCD-CT significantly reduce radiation doses while maintaining high image quality (22). The absence of consensus on preoperative imaging prior to CI emphasizes the need for standardized protocols in children. In this cohort, omission of routine preoperative CT was not associated with an observed increase in perioperative complications or duration of surgery, which could also be shown in previous studies (20). In our institution duration of surgery did not differ for experienced surgeons nor for surgeons in training, irrespective of the availability of a preoperative CT scan. This may be explained by the structured supervision at our institution, where surgeons in training discuss cases preoperatively with experienced CI surgeons and can obtain intraoperative guidance if needed. In centers without high surgical volumes, where the learning curve may be less pronounced, preoperative CT imaging could still be advantageous for surgical planning, particularly for electrode array insertion using planning software such as Otoplan (16). Nevertheless, MRI enables individual cochlear measurements comparable to CT-based evaluation and software-assisted surgical planning, although most surgeons remain more familiar with CT-based planning (15, 16, 27).

A significant difference on perioperative minor and major complications with and without CT scan prior to CI was absent, with no cases of facial nerve palsy or paralysis in both groups and one with CSF leakage with an existing preoperative CT scan. This is also consistent with previous studies (11, 20). The absence of differences in complication rates and surgical duration between patients with and without preoperative CT may however be influenced by temporal changes over the study period (2018–2024), including increasing surgical experience, changes in surgical workflows, and the use of different electrode arrays. Regarding the duration of anesthesia, the unadjusted analysis showed no significant difference between the groups, but age-adjusted analysis revealed a significantly shorter anesthesia duration in patients undergoing preoperative CT, suggesting that age adjustment may uncover an association not apparent in the unadjusted analysis.

Furthermore, our data can confirm the authors argument, that MRI can also detect relevant abnormalities and that there is a significant redundancy in information gained by the two modalities (11, 20, 26). Our findings further suggest that although preoperative CT provides additional information on bony anatomy beyond cMRI, these findings rarely influence surgical management. Among the potentially relevant CT findings, 10 were not detected on cMRI; however, only one CT-only finding resulted in a modification of the surgical procedure. This indicates and addresses the primary question of the study that the clinical value of routine CT depends on its impact on surgical planning rather than on the detection of additional abnormalities alone. Nevertheless, preoperative CT remains clinically valuable in selected patients with complex anatomy or a high risk of CSF leakage. In such cases, detailed assessment of the bony anatomy can guide the overall surgical approach, such as deciding on a subtotal petrosectomy to achieve wider exposure and cavity obliteration (28, 29).

In our study, most children showed no relevant inner ear malformations on imaging, both in the dual-imaging cohort (78.1%) and in the MRI-only cohort (83%), consistent with previous findings (21). Nevertheless, in children inner ear malformations are more present than in the adult CI candidates, particularly those with congenital sensorineural hearing loss and single sided deafness (30–33). In our cohort, inner ear malformations potentially contributing to hearing loss were identified in 21.9% of patients undergoing additional CT. This therefore implicates some cases, in which additional CT may provide relevant surgical information when complex bony anatomy is suspected. This may be particularly relevant in children with syndromic disorders such as CHARGE-syndrome or Johansson-Blizzard-syndrome or malformations detected on MRI, such as incomplete partition type (IP) III, where CT can better delineate the facial nerve course and other bony structures that may affect cochlear access and electrode insertion (11, 34). However, some studies even propose not to perform an additional CT scan of the temporal bone for several malformations, such as LVA or IP type II, the most frequent abnormalities identified on cranial imaging in their study (11). Our findings support this, as all cases of LVA and one case of IP type I were detectable on both CT and MRI, while one case of IP type II was identified only on MRI.

Although MRI may be less sensitive for certain bony features, such as mastoid sclerosis or poor pneumatization, these findings rarely alter the surgical approach or compromise CI (11, 21). Nonetheless, MRI remains indispensable prior to CI, as it allows reliable detection of cochlear nerve hypoplasia and aplasia, inner ear malformations as LVA, particularly in congenital and single-sided deafness, when an early implantation is crucial (15, 30). Overall, our findings suggest that CT should be reserved for selected patients in whom MRI or the clinical context raises suspicion of surgically relevant bony abnormalities.

In this study cohort, children without a preoperative CT scan were significantly younger. This likely reflects a change in the preoperative imaging protocol around 2021, when increasing evidence supported early implantation in children with SNHL as safe and essential for optimal long-term outcomes (6).

Intraoperative X-ray imaging was routinely performed to assess for potential tip fold-over, although none was identified. Given the need to minimize unnecessary radiation exposure, routine radiological verification warrants reconsideration. Electrophysiological methods, particularly transimpedance matrix (TIM) measurements, may reliably detect tip fold-over intraoperatively and could reduce routine imaging in appropriately selected cases, as supported by recent literature (35, 36).

## 5 Limitations

Our study is primarily limited by its retrospective, non-randomized design, which led to unequal cohort sizes and baseline disparities such as differences in age. Additionally, chronological shifts in surgical practice following the protocol change could have influenced the outcomes. Finally, although the low overall complication rate underscores the safety of the surgical procedure, the small number of adverse events restricts our statistical power. While minor complications were numerically higher in the no-CT cohort, this difference lacked statistical significance. However, because a non-significant *p*-value alone does not demonstrate equivalence, the wide confidence interval for the risk difference (−6.3 to 14.3%) indicates that a true clinical difference cannot be entirely ruled out due to our limited sample size. Additionally, the prevalence of inner ear malformations differed significantly between the CT (21.9%, 23/105) and MRI-only cohorts (1.7%, 1/59). This discrepancy potentially introduces selection and detection bias, as the lower rate in the MRI group could reflect the lower sensitivity of MRI for subtle bony abnormalities compared to temporal bone CT. Furthermore, the cohorts differed regarding patient age and the proportions of sequential versus simultaneous CI, factors known to influence duration of surgery and anesthesia as well as complications. Although we adjusted for age, residual confounding between the groups cannot be entirely ruled out.

## 6 Conclusion

Our findings support an MRI-based imaging strategy without routine preoperative CT for most pediatric CI candidates, with selective use of preoperative CT in patients with suspected complex malformations, syndromic disorders, or abnormal MRI findings requiring further bony anatomical characterization for surgical planning. However, in our study, such anomalies were also mostly identifiable on MRI and did not result in complications. Although CT provided additional information on bony anatomy in some patients, CT-only findings rarely altered surgical management.

## Data Availability

The datasets generated and/or analyzed during the current study are not publicly available due to patient confidentiality and data protection regulations but may be available from the corresponding author upon reasonable request. Requests to access these datasets should be directed to Leonie.fries@uniklinik-freiburg.de.
