# Supplementary material for: Preoperative cranial computed tomography in pediatric cochlear implantation: clinical necessity or optional step?
Source: Front Neurol. 2026 Oct 23;17:1981222. doi: 10.3389/fneur.2026.1981222 (PMC13739535; doi:10.3389/fneur.2026.1981222)
Supplement: Supplementary file 1 [file Table_1.DOCX]

Supplementary Table 1. Etiologies of sensorineural hearing loss in patients with and without preoperative CT.

| **Etiology** | **CT (n=105)** | **No CT (n=59)** |
| --- | --- | --- |
| **Congenital hearing loss** | 61 (58.1%) | 39 (66.1%) |
| **Infectious causes** | 9 (8.6%) | 5 (8.5%) |
| – Congenital CMV infection  – Meningococcal sepsis  – Rubella infection following vaccination  – Labyrinthitis following Influenza A infection | 6  1  1  1 | 4  –  –  – |
| **Genetic disorders** | 7 (6.7%) | 4 (6.8%) |
| – GJB2 mutation  – DFNB1 mutation  – LOXHD1 mutation  – Auditory synaptopathy | 4  1  1  1 | 3  –  –  1 |
| **Inner ear malformations** | 23 (21.9%) | 1 (1.7%) |
| – Semicircular canal dehiscence  – Large vestibular aqueduct (LVA)  – Cochlear dysplasia  – Neurovascular contact of the vestibulocochlear nerve  – Vestibular and inferior/lateral semicircular canal dysplasia | 12  5  5  1  – | –  –  –  –  1 |
| **Unknown etiology** | 2 (2.8%) | 6 (10.2%) |
